# Exploring Traumatic Brain Injuries and Aggressive Antisocial Behaviors in Young Male Violent Offenders

**DOI:** 10.3389/fpsyt.2020.507196

**Published:** 2020-10-09

**Authors:** Samuel Katzin, Peter Andiné, Björn Hofvander, Eva Billstedt, Märta Wallinius

**Affiliations:** ^1^Lund Clinical Research on Externalizing and Developmental Psychopathology, Child and Adolescent Psychiatry, Department of Clinical Sciences Lund, Lund University, Lund, Sweden; ^2^Centre of Ethics, Law and Mental Health, Department of Psychiatry and Neurochemistry, Institute of Neuroscience and Physiology, The Sahlgrenska Academy at University of Gothenburg, Gothenburg, Sweden; ^3^Forensic Psychiatric Clinic, Sahlgrenska University Hospital, Gothenburg, Sweden; ^4^Department of Forensic Psychiatry, National Board of Forensic Medicine, Gothenburg, Sweden; ^5^Division of Forensic Psychiatry, Trelleborg, Sweden; ^6^Gillberg Neuropsychiatry Centre, Institute of Neuroscience and Physiology, University of Gothenburg, Gothenburg, Sweden; ^7^Research Department, Regional Forensic Psychiatric Clinic, Växjö, Sweden

**Keywords:** traumatic brain injuries (TBI), violence, aggression, antisocial behavior [APA PSYNET], substance use disorder, intelligence, offender

## Abstract

**Background:** Traumatic brain injury (TBI) is a major cause of disabilities and mortality worldwide, with higher prevalence in offender populations than in the general population. Previous research has strongly advocated increased awareness of TBI in offender populations. The aim of this study was to explore the prevalence and characteristics of TBI, and to investigate associations and interactions between TBI, aggressive antisocial behaviors, general intellectual functioning, and substance use disorders (SUD) in a well-characterized group of young violent offenders.

**Methods:** The study investigated a cohort (*n* = 269) of 18 to 25-year-old male violent offenders in Sweden. Data on TBI (files + self-report), aggressive antisocial behaviors (Life History of Aggression), SUD (clinical interviews), and general intellectual functioning (General Ability Index, Wechsler Adult Intelligence Scales Third Edition) were collected between 2010 and 2012. Parametric (Student's *t*-test) and non-parametric (Mann-Whitney *U*-test, Spearman's rho, χ^2^, Kruskal Wallis test) inferential statistics were applied and effect sizes reported.

**Results:** TBI, both with and without loss of consciousness, was common, with 77.5% of the offenders reporting having suffered at least one TBI during their lifetime. TBI was associated with an increased occurrence of aggressive antisocial behaviors and SUD, and offenders with both TBI and SUD evidenced the largest amount of aggressive antisocial behaviors. No clinically meaningful associations were found between TBI and general intelligence. Effect sizes were in the small to medium range.

**Conclusions:** Our study confirms an increased prevalence of TBI among young violent offenders compared to the general population, as well as associations between TBI, aggressive antisocial behaviors, and SUD. However, it provides no information on the severity of the TBI, nor on the causality of the demonstrated associations. Nevertheless, TBI, and possible related deficits, need to be considered in the assessment and treatment of young violent offenders.

## Introduction

Traumatic brain injury (TBI) has been described as “a non-degenerative, non-congenital insult to the brain from an external mechanical force, possibly leading to permanent or temporary impairment of cognitive, physical, and psychosocial functions, with an associated diminished or altered state of consciousness” (1). Slightly different definitions have been used in the literature, often with some measurement of the severity of the injury. In one study, TBI was defined as a “blow to the head with LOC [loss of consciousness] or at least some period of being “dazed and confused” (2). The same study also classified TBI as mild if the LOC lasted <30 min, and moderate to severe if it lasted longer than 30 min. Researchers' and clinicians' attempts to develop a unitary, comprehensive definition of TBI are complicated by the fact that it is not a binary condition (3).

Regardless of the differing definitions of TBI, it has been described as the leading cause of disability and mortality in children and young adults worldwide, with possible disabilities including physical, cognitive, and mood deficits (4–7). Deficient regulation of mood and behavior, and neurocognitive problems associated with memory, attention, and executive functions are especially disabling. These deficits are predominantly associated with frontal and temporal lobe injuries (7). Deficits in metacognition, affecting insight and understanding of one's own functional limitations, have also been shown to be a major disability in individuals with TBI (8, 9). Several epidemiological studies have estimated the prevalence of TBI in the general population, with estimates of at least one TBI ranging from 13 to 20% (10, 11). In a study of lifetime occurrence of TBI, about 40% of those with some kind of TBI suffered their first TBI between ages 0 and 14, with a mean age of diagnosis of first TBI at 23 years (10). Furthermore, about 57% of the persons who had acquired a TBI were male.

It has been suggested that TBI might play a role in the development of aggressive antisocial behaviors, especially verbal aggression, and that the location (e.g., frontal lobe lesions) of the TBI might influence what type of aggression presented [i.e., physical or verbal aggression; (12)]. A population study demonstrated increased rates of violent crimes (8.8%) in individuals with TBI compared to individuals without TBI (3%) (13). In 2009, a systematic review of violence and neurological disorders demonstrated that violence was often associated with TBI while inversely associated with epilepsy (14). However, it was also pointed out that there was an overall publication bias, especially in TBI studies, and that analyses of other factors that could be related to aggression were impossible, mainly due to lack of sufficient data. Aside from studies demonstrating an association between TBI and aggressive antisocial behaviors, previous research has also demonstrated TBI occurring prior to criminal offending (15), and linked TBI to more violent crimes (2). However, it has also been demonstrated that aggressive persons with TBI already had a significantly higher level of aggressive behaviors with legal repercussions prior to their traumatic event (16).

Even if the causality in these associations remains to be established, these results illustrate the complexity of the relationship between TBI and (violent) offending and suggest that TBI might be more common in offender populations. Indeed, previous studies have shown an increased prevalence of TBI in offender populations compared to the general population, with systematic reviews reporting prevalence rates ranging from 16.5% up to 100% in specific offender populations (17, 18). A meta-analysis reported a rate of TBI with LOC of 51% among imprisoned offenders, compared to 12% in the general population (19). Among young offenders, 46% have been reported to have suffered at least one TBI with LOC, with another 19% classed as possible TBI but without LOC (19). The most common cause of TBI in incarcerated offenders and young offenders has been shown to be violence (e.g., fights), which account for ~50–60% of the TBI injuries (2, 20). Falls accounted for 10–40% of the injuries, while vehicle crashes and sports-related injuries accounted for ~30% each. In one of the studies, 78% of the offenders stated that their injuries were directly associated with their offending (20). Taken together, these findings imply that TBI, and disabilities related to TBI, should be acknowledged and managed in offender populations.

When studying TBI in offender populations, possible confounders of an association between TBI and offending need to be considered. Previous studies have demonstrated an association between TBI and varying symptoms and diagnoses of mental disorders including substance use disorders (SUD), both of which are common in offender populations (2, 21–24). A systematic review of TBI in prison populations demonstrated that comorbid mental disorders and neurocognitive deficits were more common among offenders with TBI compared to those without TBI (18). In particular, SUD needs to be considered in this respect, as an increasing number of studies show that early life TBIs increase the risk of subsequent substance use and other risky behaviors in offender populations, with younger age at TBI being associated with early substance (including alcohol) use (21, 22, 24). This seems especially important to consider in studies of the association between TBI and aggressive antisocial behaviors, since younger age at onset of substance use increases the total aggression, and there is a well-established association between SUD and violent criminality (22, 25, 26).

Another possible confounder that needs to be investigated in relation to TBI and aggressive antisocial behaviors is cognitive functioning. Several studies have shown associations between TBI and cognitive dysfunction, including problems with memory, cognitive speed, and attention deficits distinguishable early after injury in some cases, but also appearing later in some cases (27). Cognitive deficits after TBI have been reported both subjectively by the individuals themselves and on the basis of decreased scores on neurocognitive tests (28). For instance, mild TBI in preschool children has been related to subsequent lower scores on theory of mind tests compared to controls (29). In adults, the Wechsler Adult Intelligence Scales [WAIS; (30)] are often considered to correctly evaluate the effects of moderate to severe TBI on cognitive functioning (31). Even mild/moderate TBI has been associated with indices of the WAIS relating to working memory and cognitive speed, as well as Full Scale IQ (32). Furthermore, previous research indicate a negative association between intellectual functioning and aggressive antisocial behaviors, though other studies have not corroborated these findings (33–38). Thus, general intellectual functioning would need to be considered as a possible confounder.

In summary, previous research indicates the need for increased awareness of TBI in offender populations (39). The main aim of the current study is to provide increased knowledge of TBI and the impact of clinical covariates (general intellectual functioning, SUD) on TBI and the association between TBI and aggressive antisocial behaviors in a clinically well-described, nationally representative cohort of young violent offenders. The specific aims are: (1) describe the prevalence and basic characteristics of TBI in young violent offenders, (2) test associations between TBI, aggressive antisocial behaviors, general intellectual functioning, and SUD, and (3) determine interactions between prevalence of SUD, TBI, and aggressive antisocial behaviors.

## Methods

### Participants

The participants consisted of consecutively recruited male offenders aged 18–25 years who all served time between March 2010 and July 2012 at any of nine correctional facilities in the western region of the Swedish Prison and Probation Service. The offenders were sentenced for violent offenses, including hands on sexual offenses. The facilities ranged from high security to open facilities and housed ~20% of the national cohort of young, male violent offenders. Since the region had only one specialized women's prison, female offenders were excluded due to lack of statistical power for the study aims. Other exclusion criteria included poor Swedish, defined as when an interpreter would be needed for full participation, and offenders with an anticipated stay of <4 weeks at the current facility. In total, 269 offenders participated in the study, with a participation rate of 71% of those meeting the inclusion criteria. The age of the offenders ranged from 18 years and 7 months to 25 years and 11 months, with a mean age of 22.3 years (*SD* = 1.9).

In order to assess the representativeness of the sample, non-personal basic information was provided for excluded and non-consenting offenders. Those excluded because of insufficient skills in Swedish (*n* = 23) differed statistically significant from the participants by a higher rate of sexual index crimes (*n* = 12; 52%). Among the non-consenters (*n* = 109), 15 offenders (14%) had been sentenced for sexual violent crimes, and 94 offenders (86%) had been sentenced for non-sexual violent crimes. No statistically significant differences in mean age or type of index crime (general violence or sexual violence) could be seen when the non-consenters were compared to the participants. The study group, including their psychosocial background and psychiatric characteristics, is described in detail in previous publications (40–42) and is considered as nationally representative of 18–25 year old male offenders within the Swedish Prison and Probation Service who had been imprisoned due to violent crimes.

### Procedure

Offenders eligible for participation were informed of the study and provided informed, written consent prior to participation. All offenders were continuously assessed according to a preset protocol including a large number of clinical assessments in one full day. Assessors were licensed psychologists with clinical experience and specific training in the methods used. All file information from the Swedish Prison and Probation Service, including prison health care journals, reports of previous living circumstances and criminal history, and incidents while serving their sentence, was available for data collection. After meeting the participant for an entire day, the assessors concluded the preliminary assessments from all data collected (interviews, files, neurocognitive tests). Thereafter, the assessor and one of two senior clinicians and researchers with considerable experience from the field discussed all assessments and set final diagnoses and assessments together in accordance with the LEAD principle (Longitudinal, Expert, All, Data; 43).

### Measures

#### Traumatic Brain Injury

Information regarding the offenders' history of TBI was gathered through interviews with the offenders as well as through available files and medical records from the Swedish Prison and Probation Service. We did not have access to national registry data detailing information on head trauma with diagnostic codes for the current study. The final assessment was based on the information (self-report or file information, or a combination of both) that was considered credible by the assessor and the senior clinician and researcher when applying the LEAD principle (43). When information was considered not credible (e.g., due to obvious over-reporting or uncertainty in reporting) or completely missing, the values were treated as missing values. The number of participants with missing values for each of the TBI variables are reported below.

The TBI variables investigated in this study included two different types of data. The first type is the categorical variables TBI any kind (missing in *n* = 2), TBI with LOC (missing in *n* = 67), and TBI without LOC (missing in *n* = 16). The second type of data is the continuous variables number of TBI with LOC and number of TBI without LOC. Information on the age at which the participant incurred the most severe TBI (missing in *n* = 78) was also collected.

#### Aggressive Antisocial Behaviors

Lifetime aggressive antisocial behaviors were measured using the Life History of Aggression (LHA) protocol (44, 45). The LHA measures 11 different types of aggressive and antisocial behaviors with each behavior rated on a 5-point scale where 0 correlates to no behaviors occurring since adolescence, and five indicated that the behavior had occurred more times than the offender can remember or count. The LHA Total score ranges between 0 and 55. The LHA is also summed in three sub-scales: Aggression, Self-directed aggression, and Antisocial behavior. The LHA was used as a clinical assessment tool in the current study, with the assessor basing the final assessment on the information considered credible from files and interviews. For example, if the offender reported more aggressive antisocial behaviors than were noted in the files, and this information was considered credible by the assessor (e.g., not obvious over-reporting), the information from the interviews was used to score the current LHA item. In this study, only information from the three scales referring to overt (i.e., not self-directed) aggressive antisocial behaviors was used for analyses. The scale Self-directed aggression measures lifetime occurrence of self-harm and suicide attempts, which can be highly relevant in relation to both TBI and aggressive antisocial behaviors. However, this scale was not included in the current analyses since we considered this to warrant more specific analyses and focus compared to what was possible in relation to the study aims. Complete LHA data was available for 267 offenders.

#### Substance Use Disorders

The lifetime occurrence of SUD according to the DSM-IV-TR (4) was determined in consensus by the assessor and a senior clinician and researcher according to the LEAD principle (43) on the basis of interviews (Structured Clinical Interview for DSM-IV Axis I Disorders) (46) and file information. As the high prevalence of poly-substance use in this group made it difficult to make reliable decisions on withdrawal symptoms, the diagnostic categories of abuse and dependence were collapsed in this study and gathered in a binary variable; SUD/No SUD. Information on SUD was available for all offenders.

#### Intellectual Functioning

Intellectual functioning was assessed with the WAIS-III, General Ability Index (GAI) (47, 48). Assessments of intellectual functioning were available for *n* = 264 offenders. In-depth information on the intellectual and executive functioning of the offenders was previously published (38).

### Statistical Analysis

**Figure 1:**
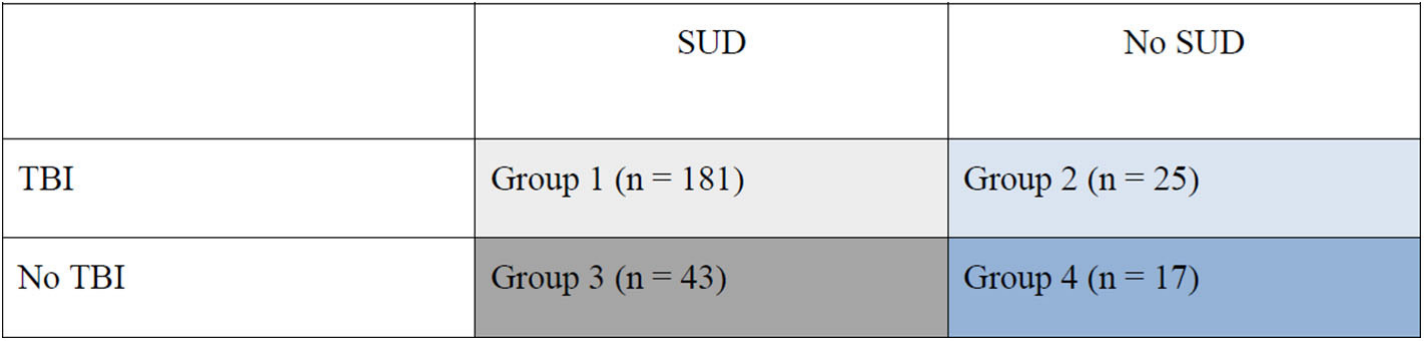
Groups of young violent offenders with or without TBI and/or SUD.

All data were anonymized and coded before being analyzed. IBM SPSS Statistics 25 was used for statistical analyses. Frequencies of TBI variables were calculated in relation to the first aim of the study. Analyses [Mann-Whitney *U*-test, Spearman's rho (*r*_*s*_), χ^2^] relating to the second aim of the study were non-parametric when testing negatively skewed data (LHA scores) and parametric (Student's *t*-test) when investigating normally distributed data (WAIS GAI scores). Effect sizes from Mann-Whitney *U*-tests were calculated using the formula *r* = Z/√n, with Φ for χ^2^ tests, and Cohen's *d* for *t*-tests. The third aim of the study was examined using a Kruskal-Wallis test with *post-hoc* comparisons between groups of offenders with or without TBI and/or SUD using Mann-Whitney U tests. Figure 1 shows the groups used. Since several offenders had missing data points for different variables, the number of offenders and the percentages presented in the results section are based on the valid percentages and those offenders available for the current analysis.

## Results

### Prevalence of Traumatic Brain Injury in Young Male Violent Offenders

Of the 269 offenders, approximately three in four had suffered at least one TBI, with the majority having suffered at least one TBI without LOC (see Table 1). That is, only 60 offenders (22.5% of the sample with information on TBI) reported no TBI. The mean number of TBI without LOC was 8.8, with a standard deviation of 20.6 (range 0–150). Of the 207 offenders who had suffered at least one TBI, more than half had suffered from TBI with LOC. The mean number of TBI with LOC was 2.3, with a standard deviation of 6.4 (range 0–60). On average, the offenders were 15.6 years old when acquiring their most serious TBI (*SD* = 4.6 years, range 2–25 years).

**Table 1:** Prevalence of traumatic brain injury and psychiatric characteristics of young male violent offenders (*n* = 269).

	***n* (%)**
**Traumatic brain injury**	
Any kind	207 (77.5)
Without LOC	187 (69.5)
With LOC	125 (46.5)
**Psychiatric characteristics**	
Any Axis I disorder	197 (73.5)
Mood disorders	144 (53.9)
Anxiety disorders	157 (51.3)
Psychotic disorders	22 (8.2)
Impulse control disorders	54 (20.3)
ADHD in childhood	169 (63.5)
ADHD in adulthood	115 (43.2)
Autism spectrum disorder	26 (9.7)
SUD	227 (84.4)
Personality disorders	176 (66.9)

*LOC, loss of consciousness. Psychiatric characteristics of the sample were previously reported (41, 42)*.

### Traumatic Brain Injury, Aggressive Antisocial Behaviors, Substance Use Disorders and Intellectual Functioning

Mann-Whitney *U*-tests showed that offenders with TBI (any kind) differed significantly on all LHA scales from offenders not reporting any TBI (Table 2). The effect sizes (*r*) were small (49). Separate analyses of offenders with TBI without LOC and those with LOC also revealed significant differences in LHA scores compared to those who had not suffered from that form of TBI. The effect sizes in these cases were small to medium. Correlation analyses demonstrated that the number of TBIs without LOC and with LOC, respectively, was correlated with all scales of the LHA with small to medium effect sizes (Table 3). The age at which the offender incurred the most severe TBI was not significantly correlated to any of the LHA scales.

**Table 2:** Associations (Mann-Whitney *U*) between traumatic brain injury and aggressive antisocial behaviors according to the Life History of Aggression.

**Traumatic brain injury**	**LHA Total**	**LHA Aggression**	**LHA Antisocial behavior**
**Any kind**			
Yes, Mdn score	34	19	14
No, Mdn score	25	14	11
*U*	3996.5	4046.5	4163.5
*p*	<0.001	<0.001	<0.001
*r*	0.26	0.25	0.24
**Without LOC**			
Yes, Mdn score	34	19	14
No, Mdn score	25	14	11
*U*	3862.5	3803.5	4083.5
*p*	<0.001	<0.001	<0.001
*r*	0.28	0.29	0.26
**With LOC**			
Yes, Mdn score	34	19	14
No, Mdn score	26	14	11
*U*	3033.5	3159.5	3283.5
*p*	<0.001	<0.001	<0.001
*r*	0.31	0.29	0.27

*LOC, loss of consciousness; LHA, Life History of Aggression; Mdn, median*.

**Table 3:** Correlations (Spearman's rho) between number of traumatic brain injuries, age at most severe traumatic brain injury, and the Life History of Aggression scales.

**Traumatic brain injury**	**LHA Total**		**LHA Aggression**		**LHA Antisocial behavior**	
	***r_***s***_***	***p***	***r_***s***_***	***p***	***r_***s***_***	***p***
Nr. without LOC	0.34	<0.001	0.33	<0.001	0.33	<0.001
Nr. with LOC	0.29	<0.001	0.27	<0.001	0.23	0.001
Age at most severe	0.04	0.554	0.05	0.464	0.02	0.777

*LOC, loss of consciousness; LHA, Life History of Aggression*.

**Figure 2:**
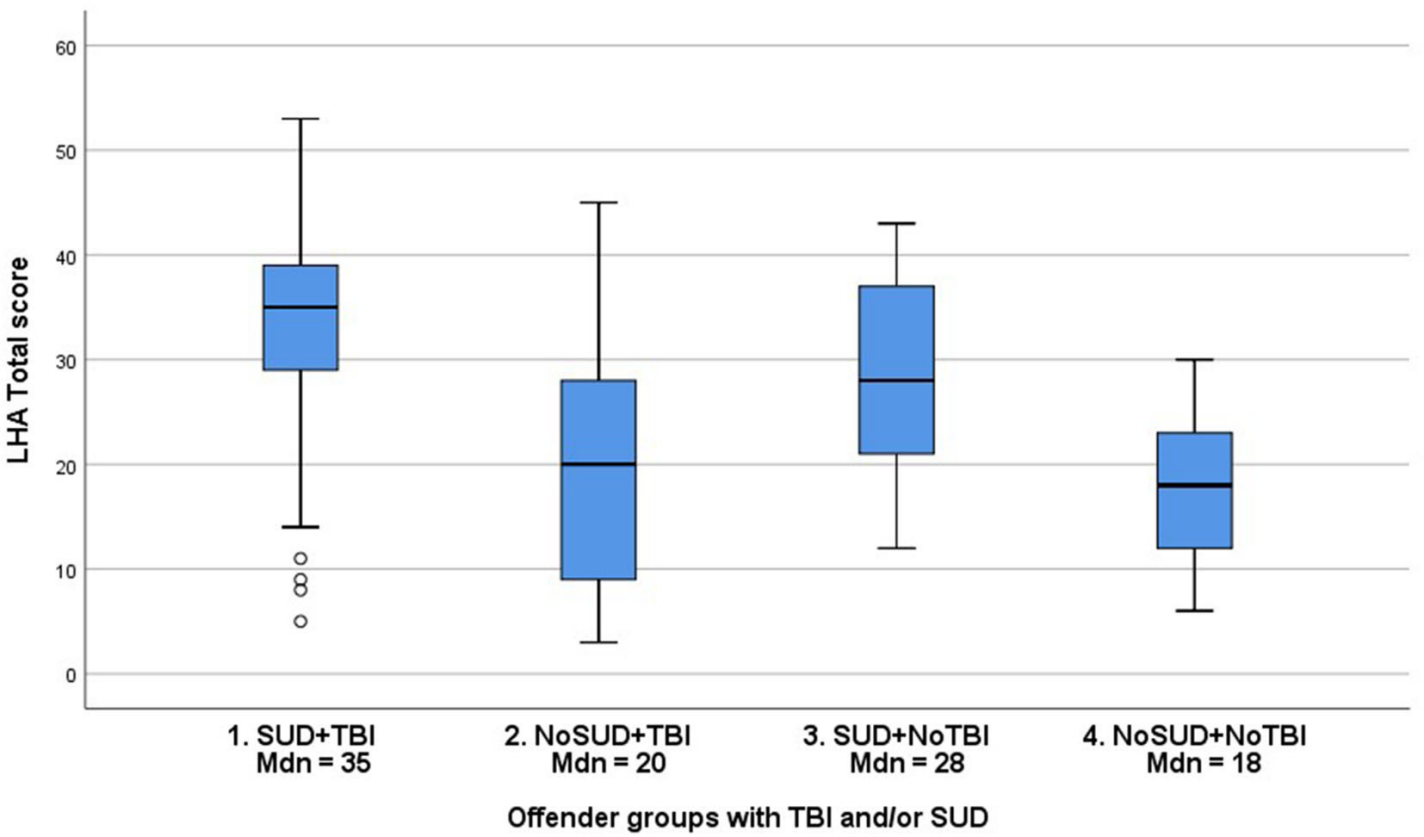
Distribution (Median, Interquartile range) of LHA Total scores in groups of young violent offenders with or without TBI and/or SUD.

As regards possible associations between SUD and TBI, χ^2^ tests demonstrated that the prevalence of SUD was significantly associated with TBI of any kind [${\text{χ}}_{\left(1,\text{n}=267\right)}^{2}$ = 8.1, *p* = 0.004, Φ = 0.19] as well as with TBI without LOC [${\text{χ}}_{\left(1,\text{n}=253\right)}^{2}$ = 7.7, *p* = 0.006, Φ = 0.19] but not with TBI with LOC [${\text{χ}}_{\left(1,\text{n}=202\right)}^{2}$ = 2.4, *p* = 0.124, Φ = 0.12]. Four groups were created to examine the influence of SUD (any kind) on the relationship between TBI and aggressive antisocial behaviors: (1) SUD and TBI; (2) No SUD but TBI; (3) SUD but No TBI; (4) neither SUD nor TBI (see Figure 1). Kruskal-Wallis test was used, demonstrating a statistically significant difference in LHA Total scores across the four groups, *H*_(3,*n* = 266)_ = 52.3, *p* < 0.001. *Post-hoc* tests revealed statistically significant differences between all groups (1–4), 0.001 ≤ *p* ≤ *0.0*07, except between the groups with no SUD (groups 2 and 4). In general, the SUD groups (groups 1 and 3) demonstrated higher LHA Total scores. Effect sizes were in the small to medium range (49), 0.20 ≥ *r* ≤ 0.46, with the smallest effect size for the comparison between groups 1 and 3 and the largest effect size for the differences between groups 3 and 4 (see Figures 1, 2 for group illustrations).

When TBI was investigated in relation to the offenders' intellectual functioning, offenders with any kind of TBI had statistically significant higher WAIS GAI scores than offenders without any kind of TBI (Table 4). The same pattern was found for offenders who had incurred at least one TBI without LOC, but not for offenders with TBI with LOC. Note that the effect sizes were very small (49), and that in practice the differences in GAI scores between offenders with and without TBI were smaller than one third of a *SD* (15 points) according to the WAIS-III manual (47).

**Table 4:** Associations (Student's *t*-test) between traumatic brain injury and intellectual functioning.

**Traumatic brain injury**	**WAIS-III GAI**
**Any kind**	
Yes, M (*SD*)	94.6 (10.8)
No, M (*SD*)	90.0 (11.1)
*t*	−2.93
*p*	0.004
*d*	0.04
**Without LOC**	
Yes, M (*SD*)	94.7 (10.8)
No, M (*SD*)	90.2 (10.7)
*t*	−2.86
*p*	0.005
*d*	0.04
**With LOC**	
Yes, M (*SD*)	94.4 (10.5)
No, M (*SD*)	92.0 (11.6)
*t*	−1.49
*p*	0.138
*d*	N/A

*LOC, loss of consciousness; M, mean; SD, standard deviation; WAIS-III, Wechsler Adult Intelligence Scales, Third Edition; GAI, General Ability Index*.

## Discussion

The aims of this study were to characterize TBI and to determine associations and interactions between TBI, general intellectual functioning, and SUD in a nationally representative and clinically well-described cohort of young violent offenders in Sweden. TBI was very common in the young offenders; approximately three in four reported having suffered at least one TBI during their lifetime, with a high prevalence of both TBI with and without LOC. TBI was associated with higher levels of aggressive antisocial behaviors and higher prevalence of SUD. Furthermore, an interaction between SUD and TBI was noted in relation to aggressive antisocial behaviors. However, all effect sizes were in the small to medium range, and any interpretation of the findings must take cognizance of the methodological limitations of this study due to information on TBI being gathered from files and self-report from the offenders.

The prevalence of TBI in this group of young, violent offenders was 3–6 times higher than estimates for the general population (10, 11). This is in line with previous studies on offender populations, even though previous estimates vary with the specific offender population studied (17, 18, 20, 50). The most common type of TBI was without LOC, with 69.5% of the whole cohort (90% of the total TBI group) having incurred at least one TBI without LOC during the lifetime. However, TBI with LOC was also common, with a lifetime prevalence of 46.5% in the whole group and 60.4% in the total TBI group. This is in line with previous findings of TBI with LOC rates at 40–50% in offender populations (18, 19, 50). These results imply that the findings are more likely to be representative for the studied group and that further conclusions might represent not only the studied group, but also groups in similar conditions.

The current study does not provide any answers about the possible causes of TBI in young violent offenders since this was not studied. The general lifestyle and psychiatric characteristics of these young men shows a high prevalence of, and variation in, criminal activities with an early onset as well as a high prevalence of ADHD, antisocial personality disorder, and SUD (40–42). It seems probable that previous research, showing TBI resulting from a criminal lifestyle (e.g., assaults) may also apply to the currently studied group (2, 20). In that sense, it seems likely that they are not only perpetrators of violence against others but are also subjected to violence themselves, and that both their lifestyle and psychiatric characteristics need to be considered when interpreting possible causes of TBI in these groups.

Our results demonstrate that the young offenders had incurred TBIs on several different occasions, with a mean number of TBI without LOC of 8.8 (range 0–150) and mean number of TBI with LOC of 2.3 (range 0–60). Even though the ecological validity of these findings can be questioned due to study's reliance on self-report and files, the findings are in line with previous research demonstrating multiple TBIs in offenders and stresses the importance of assessing and treating multiple injuries in offenders since multiple milder injuries can result in similar cognitive and behavioral challenges as more severe, singular injuries (20, 51–53). However, our results need to be interpreted with care since the reliability of a young man with a criminal lifestyle and substance use problems remembering 150 specific occasions of TBI might be questioned. Data such as these would need to be corroborated by other, objective data such as care seeking behavior related to TBI. However, due to the characteristics of these groups, which include a general reluctance to seek care, it is not certain that such data could provide a clearer picture, especially since seeking care for TBI could compromise their criminal lifestyle by alerting the police to violent crimes (54). These matters remain to be investigated.

Aside from having incurred many TBIs, the offenders reported a young age (*M* = 15.6 years, range 2–25 years) when acquiring their most serious TBI. It would seem probable that this could be related to an early onset criminal lifestyle and psychiatric comorbidity such as ADHD and early onset SUD, as has been demonstrated in this and other groups of offenders (40–42, 55). However, since we did not use official records or data from social services when conducting the study, we cannot provide any more information as to how, why, and under what circumstances these TBIs occurred. One conclusion that can be drawn is that TBI needs to be considered not only in adult offenders but also in young offenders, as it is likely to affect their clinical presentation and treatability in forensic settings.

In Mann-Whitney *U*-tests, significant differences were demonstrated in all LHA scales between offenders who had suffered at least one TBI (all variables) and those who had not were demonstrated, with small to medium effect sizes. Taken together, this is consistent with the general consensus that TBI is positively associated with aggressive antisocial behaviors (12, 13, 22, 56, 57). The age at which the most severe TBI occurred did not, however, show any significant correlations with the LHA scores. When interpreting the results, the actual differences in the LHA scale scores need to be considered. The median LHA Total score of those who had suffered a TBI (any kind) and those who had not were 34 and 25, respectively. As the LHA measures 11 items with a maximum score of five for each item, the group difference in LHA Total scores signifies approximately one point higher on all LHA items, across all offenders. Similar levels of differences were found for the other LHA scales measured in this study. The clinical significance of a one-point difference in LHA scores means the severity level of aggressive antisocial behaviors increased one level. In the real world, this difference could be clinically meaningful in separating more aggressive antisocial individuals from somewhat less aggressive antisocial individuals, even though the demonstrated effect sizes were small to medium.

It is important to note that a study of a possible association between TBI and aggressive antisocial behaviors is not the same as a study of a possible association between TBI and offenses. Though previous studies have demonstrated associations between TBI and offenses (17–19), other studies have shown that aggressive patients with TBI had a significantly higher level of aggressive behaviors with legal repercussions already prior to their traumatic event (16). Though both imply an association between TBI and offenses, there are still uncertainties regarding cause and effect. Further studies applying longitudinal design with multi-method measures of TBI, aggressive antisocial behaviors, and important confounders are needed. Studies investigating interactions and possible moderators or mediators of the association between TBI and aggressive antisocial behaviors, such as impulsivity, emotion regulation, or sociodemographic factors, are also called for since previous research has shown that problems with inhibition of destructive behaviors (e.g., binge drinking) might be associated with TBI (58).

SUD, previously demonstrated as highly prevalent in the current study group (84%) and other offender groups, has been strongly related to aggressive antisocial behaviors (25, 26, 42, 59, 60). In the current study, 181 offenders (67.3%) had SUD and had suffered at least one TBI at some point during their lifetime, showing a large overlap between TBI and SUD. Our analyses demonstrated statistically significant associations between the TBI variables and SUD. However, the effect sizes were very small (0.12 ≥ Φ ≤ 0.19). Based on these findings, it is premature to draw any conclusions regarding the relationship between TBI and SUD in young, violent offenders, since the data provided little variation with a high prevalence of both SUD and TBI in the studied group. However, our investigation of interactions between SUD, TBI, and aggressive antisocial behaviors demonstrated statistically significant differences between all groups of offenders with or without TBI and/or SUD except between the groups with no SUD (see Figure 2). The group of offenders with both TBI and SUD had the highest median LHA total scores (35), while the offenders with neither TBI nor SUD had the lowest median LHA total scores (18), corresponding to half the scores of the TBI + SUD group. Even though the effect sizes were small to medium, the actual differences in LHA scores are clinically meaningful since they correspond to a 1–2 level difference in severity of aggressive antisocial behaviors. Our findings imply an interaction between TBI and SUD in relation to aggressive antisocial behaviors in young violent offenders that needs to be investigated in future research, especially concerning moderation and/or mediation effects (53).

When TBI was investigated in relation to the offenders' intellectual functioning, higher WAIS GAI scores were found both for offenders with TBI (any kind) and offenders with TBI with LOC. Even though these differences were statistically significant, the effect sizes and inspection of the differences in mean values (~4 points on the WAIS GAI) indicate that these differences are negligible. Thus, in this group of young violent offenders, general intellectual functioning was not related to TBI in a clinically meaningful way. Since the GAI consists of the verbal comprehension and perceptual reasoning indices of the WAIS, which are generally considered to be less affected by TBI, it seems plausible that the GAI is more suitable for evaluation of pre-TBI intellectual functioning (31). However, one possible, albeit speculative, explanation of our results could be that the methods used for data collection (self-report in combination with file reviews, but no official register data) affected the results. Offenders with lower general intellectual functioning might be underreporting TBI due to cognitive deficits affecting their perception and memory of traumatic events. Furthermore, since we studied a specific population of young violent offenders with a high prevalence of neurodevelopmental disorders (41), we must consider sample bias and uninvestigated neurophysiological and/or neuropsychological characteristics as possible confounders. In sum, our results on the associations between TBI and intellectual functioning must be considered preliminary. These associations need to be investigated with other study designs, that may be more sensitive to TBI in similar samples before any conclusions can be drawn. Longitudinal, clinical investigations including other measures of intellectual proficiency (e.g., working memory, attention, cognitive speed) may be suitable.

The current study is limited by the complex characteristics of the studied group, with a high prevalence of TBI, SUD, aggressive antisocial behaviors, mental disorders, and adverse childhood experiences (40–42). Since a large proportion of the group had both TBI and SUD, there was little variation to study in the analyses. However, in-depth studies on offenders require substantial resources and are rare. Thus, our results can be used as guidance for future studies with designs that take these limitations into consideration. Another limitation is that information on TBI was gathered from files and self-reports, with no available files from care settings where a TBI could have been formally investigated (e.g., with functional measures of brain activity) and registered. The reliability and ecological validity of the TBI variables, especially the continuous variables (number of TBI, age at most severe TBI), can thus be questioned. Unfortunately, we did not have access to data regarding the severity of the TBI (e.g., type of concussion, lesions, duration of LOC) that would have made more fine-grained analyses and conclusions possible. Obviously, this needs to be considered in future studies, and more information gathered on the circumstances and severity of the TBI to increase the knowledge of TBI in young violent offenders. However, due to the studied groups general reluctance to seek help, these limitations are shared with the majority of previous publications on TBI in offenders. In future studies, it would be of value to test the validity of self-reported TBI in comparison to register information on TBI in offender groups, since both methods have inherent limitations. Also, future studies should investigate self-directed aggression in relation to TBI, since this was not analyzed within the current study. Finally, due to the cross-sectional design of the study, with some variables only available in binary form and other very skewed, we could not investigate causal effects or go deeper into interaction effects. Considering all these limitations, our study should be considered as exploratory, laying the groundwork for longitudinal studies of offenders. However, despite its methodological limitations, the current study also has several strengths that give it scientific value, specifically the use of a nationally representative cohort and the thorough clinical assessments of SUD and general intellectual functioning.

## Conclusion

Young violent offenders have an increased prevalence of TBI compared to the general population. Despite the limitations of the TBI data collected in the current study (retrospectively gathered data lacking information on type of injury etc.), TBI and thereto possible related deficits need to be considered in the assessment and treatment of young violent offenders. It seems especially important to consider these factors when planning interventions and rehabilitation to society, since cognitive deficits related to TBI can affect offenders' treatability. A recent study examined the awareness of TBI in probation services staff, revealing profound knowledge gaps concerning TBI (39). Especially worrying was observed over-reliance on offenders' self-awareness of their TBI, something the authors concluded may pose significant barriers to rehabilitation of offenders.

Our results strengthen previous findings that TBI is associated with aggressive antisocial behaviors, but no new knowledge on the causality of such a relationship is provided. We also do not know how the location of the injury, subtype of aggression or a wider spectrum of intellectual functioning (e.g., assessed using the full WAIS, or an equivalent, instead of the GAI) might affect the results, why this might be vital in further studies. However, regardless of whether TBI does or does not cause aggressive antisocial behaviors, it is important to quickly identify and monitor children and young adults who suffer TBIs, to prevent the possible development of such behavior and mitigate other deficiencies related to the TBI. The primary recovery after TBI occurs during the 2 years following the injury, but the process of recovery after that is not equally well-understood (61). Identifying those with TBI and intervening during the early stages of recovery might prove vital in preventing a development of destructive behaviors and improving the quality of life of those affected, while also resulting in more efficient use of economic resources.

## Data Availability Statement

The datasets generated for this study are available on request to the corresponding author.

## Ethics Statement

Participation in the study was voluntary, with offenders providing written, informed consent prior to participation. All offenders were offered the possibility of receiving feedback on the preliminary results of the assessments at the end of the meeting with the assessor. Those offenders who showed signs of severe psychopathology also had the opportunity to be referred to the prison physician (psychiatrist, if available) for continued evaluation and treatment. After participation, the offenders were given a SEK 200 payment, as reimbursement for the time spent in the study. The study and the monetary reward were approved by the Research Ethics Committee at Lund University, Dnr. 2009/405.

## Author Contributions

SK, MW, PA, and BH conceived and designed the study. EB, MW, and BH obtained the data, and SK, BH, and EB controlled the data. SK and MW did all the analyses and drafted the initial manuscript. Finally, all authors critically revised the manuscript and approved the final version.

## Conflict of Interest

The authors declare that the research was conducted in the absence of any commercial or financial relationships that could be construed as a potential conflict of interest.
